# Modulating arousal to overcome gait impairments in Parkinson’s disease: how the noradrenergic system may act as a double-edged sword

**DOI:** 10.1186/s40035-023-00347-z

**Published:** 2023-03-26

**Authors:** Anouk Tosserams, Bastiaan R. Bloem, Kaylena A. Ehgoetz Martens, Rick C. Helmich, Roy P. C. Kessels, James M. Shine, Natasha L. Taylor, Gabriel Wainstein, Simon J. G. Lewis, Jorik Nonnekes

**Affiliations:** 1 Department of Neurology, Center of Expertise for Parkinson and Movement Disorders, Radboud University Medical Centre, Donders Institute for Brain, Cognition and Behaviour, Nijmegen, The Netherlands; 2 Department of Rehabilitation, Center of Expertise for Parkinson and Movement Disorders, Radboud University Medical Centre, Donders Institute for Brain, Cognition and Behaviour, PO Box 9101, 6500 HB Nijmegen, The Netherlands; 3 Department of Kinesiology and Health Sciences, University of Waterloo, Waterloo, ON Canada; 4 Department of Neuropsychology and Rehabilitation Psychology, Donders Institute for Brain, Cognition and Behaviour, Radboud University, Nijmegen, The Netherlands; 5 Department of Medical Psychology and Radboudumc Alzheimer Center, Radboud University Medical Centre, Donders Institute for Brain, Cognition and Behaviour, Nijmegen, The Netherlands; 6 Vincent Van Gogh Institute for Psychiatry, Venray, The Netherlands; 7 Klimmendaal Rehabilitation Center, Arnhem, The Netherlands; 8 Brain and Mind Centre, Parkinson’s Disease Research Clinic, School of Medical Sciences, University of Sydney, Camperdown, NSW Australia; 9 Centre for Complex Systems, The University of Sydney, Camperdown, NSW Australia; 10 Department of Rehabilitation, Sint Maartenskliniek, Nijmegen, The Netherlands

**Keywords:** Arousal, Gait, Parkinson’s disease, Freezing of gait, Locus coeruleus

## Abstract

In stressful or anxiety-provoking situations, most people with Parkinson’s disease (PD) experience a general worsening of motor symptoms, including their gait impairments. However, a proportion of patients actually report benefits from experiencing—or even purposely inducing—stressful or high-arousal situations. Using data from a large-scale international survey study among 4324 people with PD and gait impairments within the online Fox Insight (USA) and ParkinsonNEXT (NL) cohorts, we demonstrate that individuals with PD deploy an array of mental state alteration strategies to cope with their gait impairment. Crucially, these strategies differ along an axis of arousal—some act to heighten, whereas others diminish, overall sympathetic tone. Together, our observations suggest that arousal may act as a double-edged sword for gait control in PD. We propose a theoretical, neurobiological framework to explain why heightened arousal can have detrimental effects on the occurrence and severity of gait impairments in some individuals, while alleviating them in others. Specifically, we postulate that this seemingly contradictory phenomenon is explained by the inherent features of the ascending arousal system: namely, that arousal is related to task performance by an inverted u-shaped curve (the so-called Yerkes and Dodson relationship). We propose that the noradrenergic locus coeruleus plays an important role in modulating PD symptom severity and expression, by regulating arousal and by mediating network-level functional integration across the brain. The ability of the locus coeruleus to facilitate dynamic ‘cross-talk’ between distinct, otherwise largely segregated brain regions may facilitate the necessary cerebral compensation for gait impairments in PD. In the presence of suboptimal arousal, compensatory networks may be too segregated to allow for adequate compensation. Conversely, with supraoptimal arousal, increased cross-talk between competing inputs of these complementary networks may emerge and become dysfunctional. Because the locus coeruleus degenerates with disease progression, finetuning of this delicate balance becomes increasingly difficult, heightening the need for mental strategies to self-modulate arousal and facilitate shifting from a sub- or supraoptimal state of arousal to improve gait performance. Recognition of this underlying mechanism emphasises the importance of PD-specific rehabilitation strategies to alleviate gait disability.

## Introduction

A person’s mental state is an important intrinsic factor affecting the expression of a variety of neurological motor symptoms, from dystonia to hemiparesis after stroke [1]. Parkinson’s disease (PD) is a prototypical example highlighting this captivating interaction, where alterations in the mental state often lead to immediate observable changes in motor symptoms. While tremor is the prime example of a PD motor feature that typically worsens during stressful situations and with increased cognitive load [2–6], other motor symptoms, such as gait impairments, are also often aggravated by stress and anxiety [6–8]. Gait impairments in PD comprise both continuously present deficits (e.g., reduced step length and height, reduced gait speed, and increased gait variability) as well as episodic deficits (i.e., festination and freezing of gait) [9]. The detrimental effect of stressful, anxiety-inducing situations on the occurrence and severity of freezing of gait episodes has been particularly well-established [10–16]. Unsurprisingly, many people with PD and gait impairment use strategies to counteract stress or anxiety, in an effort to improve their walking ability. Of these approaches, mindfulness-based interventions have received the most attention in recent years [17–20].

Paradoxically, in contrast to this negative impact on gait, some PD patients seem to actually benefit from experiencing—or even purposely inducing—stressful situations. The most extreme form is the well-known phenomenon of ‘kinesia paradoxa’: a sudden, transient ability to perform a task that a person was previously unable to complete, often in the context of grave, life-threatening situations [21, 22]. A classic case example was offered by a group of 14 institutionalized Italian patients with advanced PD, who demonstrated an extraordinary motor improvement during the L’Aquila earthquake of 2009 [23]. All of them were able to escape unaided from the collapsing nursing home, despite usually requiring assistance during daily activities because of severe gait difficulties and postural instability. While kinesia paradoxa may well be an entity on its own, it has been hypothesized that gait improvement under more mundane circumstances that increase motivation may share a similar underlying mechanism, namely a shift to optimally heighten arousal [24]. These common clinical observations raise an interesting question—how is it that heightened arousal can augment symptoms in some individuals, while alleviating their severity in others?

**Fig. 1:**
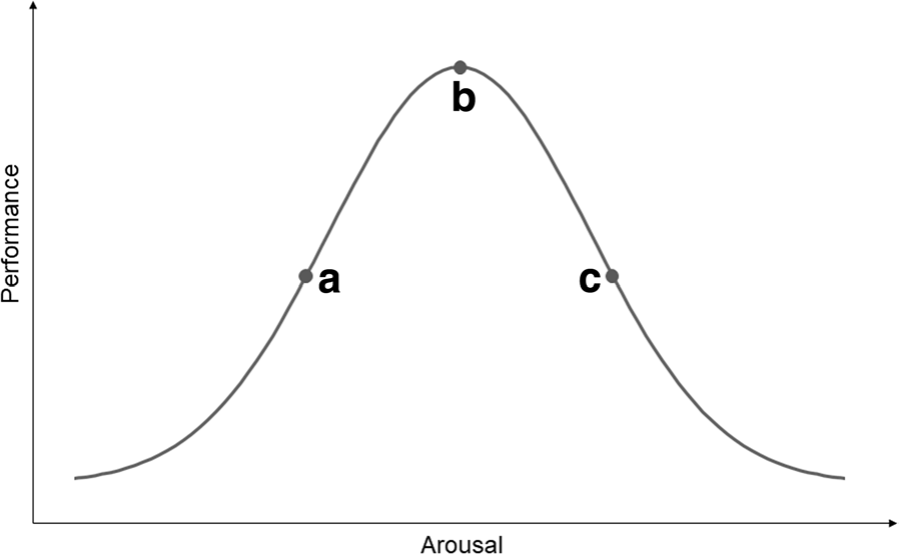
The relationship between arousal and task performance, related to the effects of compensatory strategies targeting arousal. Based on Yerkes and Dodson [25]. (a) Suboptimal state of arousal. In the case of suboptimal arousal, a person with Parkinson’s disease (PD) would likely benefit from applying a compensation strategy that aims to increase the level of arousal (e.g., by adding an element of time pressure), in order to optimize task performance. (b) Optimal state of arousal. In the case of optimal arousal, optimal task performance is expected. (c) Supraoptimal state of arousal. In the case of supraoptimal arousal, a person with PD would likely benefit from applying a compensation strategy that aims to reduce the level of arousal (e.g., by employing relaxation techniques), in order to optimize task performance

Here, we suggest that this paradox is related to inherent features of the ascending arousal system. Over a century ago, psychologists Yerkes and Dodson demonstrated that task performance is related to arousal (Fig. 1) [25]. The inverted u-shaped curve illustrates that task performance improves with increasing arousal, until an optimum is reached. When the optimal level of arousal is surpassed, performance declines again as arousal increases further. Depending on where you are situated on this curve, a change in arousal could therefore be either beneficial or detrimental to performance of the task at hand. Translating this notion to gait impairments in PD, on the left-hand side of this curve (i.e., with suboptimal arousal), gait might be ameliorated by strategies that increase arousal levels. Conversely, on the right-hand side of the curve (i.e., with supraoptimal arousal) gait might benefit from strategies that decrease arousal.

Compensation strategies tapping into this mechanism of modulating arousal levels are typically attributed to a category entitled ‘Altering the Mental State’. This is one of seven major categories of compensation strategies for gait impairment, based on a comprehensive review of several hundred videos of persons with PD who spontaneously ‘invented’ strategies to improve their gait [24]. The ‘Altering the Mental State’ category has thus far received relatively little attention, certainly when compared to other categories such as external or internal cueing [26]. For obvious reasons, it is not recommended to implement life-threatening situations in daily life to improve gait impairment. However, reports on ‘everyday’ variants of less drastic arousal strategies are scarce. Indeed, PD healthcare professionals rarely apply strategies from the ‘Altering the Mental State’ category in their clinical practice [27], even though persons with PD find it a valuable way to cope with their gait impairments in daily life [28]. Furthermore, the mechanisms underlying this category of strategies remain poorly understood.

Here, we introduce a novel theoretical framework regarding the potential underlying mechanisms of modulating one’s arousal to optimize gait performance in PD. To this aim, we first present an array of ready-to-use ‘Altering the Mental State’ strategies for gait impairment, informed by data from a large-scale international survey study among over 4000 persons with PD and gait impairments [28], illustrating our hypothesis that the noradrenergic system may act as a double-edged sword in PD gait control.

## Methods

### Study population

The present study is a secondary analysis of a web-based survey that was distributed among 6700 participants within the Fox Insight cohort (USA), as well as 1573 Dutch participants within the ParkinsonNEXT cohort (NL) [28]. Fox Insight is a longitudinal, virtual, patient-centered observational study on PD led by the Michael J Fox Foundation. Data used in the preparation of this article were obtained from the Fox Insight database on June 1st 2020. For up-to-date information on the study, visit https://foxinsights-info.michaeljfox.org/insight/explore/insight.jsp. ParkinsonNEXT (http://www.parkinsonnext.nl) is an online platform that aims to unite patients, researchers and clinicians wanting to contribute to research and innovation in PD or parkinsonism. The online survey was accessible from March to June 2020. Respondents above the age of 18 years with a self-reported diagnosis of PD and self-reported disabling gait impairments were included in the analyses.

### Survey

Details on the design and content of the original survey, which consisted of three parts, have been previously reported [28]. We will reiterate the elements relevant to the present study. The survey addressed seven main categories of compensation strategies, including ‘Altering the Mental State’ [24]. This category was explained and illustrated by several practical examples. Participants were then queried whether they had ever applied a strategy belonging to the ‘Altering the Mental State’ category, and—if so—what specific strategy they had used (free-text entry).

### Data processing and analysis

Based on the free-text entries that respondents had provided, data were verified and manually corrected by two independent researchers, to ensure that all recorded compensation strategies were completed under the appropriate corresponding category. ‘Altering the Mental State’ strategies were then classified into being either ‘strategies that reduce arousal’ (e.g., relaxation techniques, mindfulness) or ‘strategies that increase arousal’ (e.g., experiencing high-pressure situations, getting angry). Strategies that were difficult to intuitively place into one of two categories were discussed among all study investigators until consensus was reached. All (descriptive) statistical analyses were performed in IBM SPSS 25 (SPSS, Inc., Chicago, IL). Independent *t*-tests and chi-square tests were performed to assess group differences in demographic characteristics. Values of *P* < 0.05 were considered to be statistically significant.

### Ethical approval

The study was approved by the Institutional Review Board of the Radboud University Medical Center in Nijmegen, the Netherlands (Ref: 2019-5737). Written informed consent was not necessary for this work.

## Results

### Study population

In total, 5832 respondents successfully completed the questionnaire. We collected 4987 responses via Fox Insight (response rate: 74.4%), and 845 via ParkinsonNEXT (response rate: 53.7%), of which 1508 persons were excluded as they did not experience disabling gait impairments. Characteristics of the remaining final sample of 4324 respondents, of whom 1343 (31.1%) reported to have ever used ‘Altering the Mental State’ strategies, are presented in Table 1*.* Notably, the sample of respondents who used ‘Altering the Mental State’ strategies comprised relatively more women (47.4% vs 43.6%, *P* = 0.022), and the prevalence of freezing of gait (49.2% vs 41.6%, *P* < 0.001) and falls (58.4% vs 49.7%, *P* < 0.001) was higher in this group compared to the sample of respondents who did not use ‘Altering the Mental State’ strategies.

**Table 1:** Respondent characteristics

	Total cohort	Have used ‘Altering the Mental State’ strategies	Have never used ‘altering the mental state’ strategies	*P*-value^b^
Respondents (*n* (%))	4324	1343 (31.1)	2981 (68.9)	
Men (*n* (%))	2387 (55.3)	706 (52.6)	1681 (56.4)	0.022*
Age (years)	67.8 ± 9.0	67.5 ± 9.0	68.6 ± 9.0	0.371
Time since diagnosis (years)	6.7 ± 5.3	7.0 ± 5.3	6.5 ± 5.2	0.004*
Respondents with FOG (*n* (%))	1900 (43.9)	661 (49.2)	1239 (41.6)	< 0.001*
NFOG-Q score^a^ (median [range])	17 [1–28]	18 [1–27]	17 [1–28]	0.005*
Experienced ≥ 1 fall in preceding 12 months (*n* (%))	2266 (52.4)	784 (58.4)	1482 (49.7)	< 0.001*

Values are presented as mean ± SD, unless otherwise specified

FOG, Freezing of gait; NFOG-Q,  New Freezing of Gait Questionnaire (score range 0–28) [29]

The asterisks indicates that the presented* p*-value has surpassed the threshold of statistical significance (<0.05)

^a^Among respondents with freezing of gait, defined by a non-zero NFOG-Q score

^b^Respondents who had ever used ‘Altering the Mental State’ strategies versus Respondents who had never used ‘Altering the mental State’ strategies, assessed by independent *t*-tests and chi-square tests

*Statistically significant difference

### ‘Altering the Mental State’ strategies

As expected, we were able to identify a clear divide between those strategies that seemed to reduce arousal (i.e., through reducing stress or anxiety) at one end of the spectrum versus those strategies aimed at purposefully increasing arousal (i.e., through increasing stress levels or motivation) at the other end. An overview of the specific ‘Altering the Mental State’ strategies that the 1343 respondents employed to overcome their gait impairments in daily life is presented in Table 2. Some of the examples represent strategies that include a clear element of ‘Altering the Mental State’ in combination with elements of other known categories of compensation (e.g., motor imagery or internal cueing) [24].

**Table 2:** Reported ‘Altering the Mental State’ strategies for gait impairments in Parkinson’s disease

Principle mechanism	Phenomenology	Examples
Reducing arousal	Facilitating general relaxation	Mindfulness Breathing techniques Meditation Yoga Qigong Tai chi Self-hypnosis Spirituality Low-intensity physical exercise* Doing something one loves Being on holiday Being somewhere one loves Listening to one’s favourite music Taking anxiolytic drugs, or medicinal cannabis
	Eliminating negative emotions and cognitions surrounding gait	Focussing on what you CAN do Rationalize stressful events Consciously stop worrying Thinking about one’s most positive experiences Using mantra’s, or positive affirmations Visualizing a successful situation Taking antidepressants
	Decreasing external (social) pressure	Avoiding feeling rushed by other persons Communicating beforehand how one is feeling, so people can take it into account Pretending to be the only person around
	Having a back-up plan in case of gait difficulties	Carefully planning out the walk beforehand ‘Crisis rehearsal’ of bottleneck areas of the route beforehand Walking a ‘test round’ indoors before heading outside Wearing laser shoes, without having to look at the projections Holding a cane, without using it for support Having someone close by
Increasing arousal	Internal factors	Getting angry at oneself and using that energy to walk Inflicting pain on oneself Getting ‘pumped’ through forceful self-talk or high-intensity physical exercise* Purposefully creating a time-pressure situation Pretending to be on stage, ready to perform in front of a large audience Challenging oneself to make each step better than the one before
	External factors	Being in ‘test’ situations, such as at the doctor’s office Being in an emergency, or otherwise thrilling situation

*While low-intensity exercise is typically applied to facilitate general relaxation (i.e., decrease arousal), some persons employ higher intensity physical exercise to ‘get pumped’ (i.e., increase arousal)

#### Strategies that reduce arousal

Among all reported ‘Altering the Mental State’ strategies, strategies to reduce arousal were most common (76.5%). They most frequently entailed: breathing exercises (*n* = 593); mindfulness and meditation (*n* = 522); stretching before walking (*n* = 190); and praying (*n* = 31). Respondents reported many creative ways to achieve a sense of general relaxation to improve gait before going out for a walk, ranging from listening to soothing music to playing a game of digital solitaire before going anywhere: *“I recite the 7 countries of Central America and the 13 countries of South America, this relaxes me. I’m learning Europe next”.* Others reported to specifically focus on tackling negative emotions or cognitions surrounding gait right before or during walking: *"I have always been a believer in the individual’s ability to get more from their mental attitude than they otherwise do. I focus on the mental reasons I freeze, such as a fear of falling, and tell myself quite profoundly that it is only a fear and I must, simply must overcome it”.* Some respondents reported to walk better when they feel like they are well-prepared and have a back-up plan in case gait difficulties (e.g., freezing of gait) emerge: “*I always take a ‘test walk’ in the family room before going outside”; “I always carry around a cane, even though I do not actually use it for support. Just having it with me gives me more confidence to walk, as I will have something to help me in case I freeze. I feel like I freeze more often when I do not bring the cane with me.”*

#### Strategies that increase arousal

Strategies that seemed to purposefully increase arousal (23.5%) most often comprised: getting oneself angry or ‘pumped’ (*n* = 59) and forceful (often abusive) self-talk to motivate oneself to move (*n* = 86). Multiple respondents mentioned that they had noticed a marked improvement in their ability to move when they experienced stress or ‘a rush of adrenalin’: *“When my adrenaline is high, I am suddenly able to perform actions that I could not do before”; “Stress increases my focus and the fear factor makes me perform and walk better”*. Some indicated that they would simulate this feeling by getting themselves very angry: *“When I get very angry I feel my power increasing, it even enables me to run”;* or by challenging themselves: *“If I imagine a twisty trail with rocks and roots and other obstacles while walking, I'm more alert and I walk better”*. Another respondent even reported to purposefully inflict pain on herself to achieve an improvement in gait*: “I find that if I slap my right wrist really hard with my left hand, that for the next hour or more, my mind is confused and will concentrate on the wrist pain and forget the leg issues”.*

In addition to these examples of inducing arousal with negative valence, high arousal states with positive valence were reported to improve gait performance in a similar matter. Specifically, respondents mentioned to benefit from inducing a state of ‘flow’ (a state of being fully absorpted by a task, established by an optimal match between the person’s skills and the task challenges [30]) through doing something they enjoyed: *“After an intense programming session on my computer, I found myself being ‘freed’ of my Parkinson’s disease for several hours”.* Physical exercise before walking was also frequently reported to improve overall gait performance. Physical activity is a special example of ‘Altering the Mental State’ strategies, as it can be employed to facilitate general relaxation with low-intensity exercises (i.e., a decrease in arousal), as well as induce general excitement with higher intensity exercises (i.e., an increase in arousal). While some respondents reported to prefer to relax through yoga or tai chi exercises, others preferred to exercise in order to ‘kickstart’ themselves, for example by performing a quick set of push-ups before going out for a walk.

### Subgroup characterization

A characterization of the subgroups of respondents based on the type of strategies they had ever tried is presented in Table 3. Most respondents had only ever tried strategies aimed at reducing arousal (83.5%). Notably, the subgroup of respondents that had used strategies to increase arousal comprised more men (65.2% vs 52.6%) and more individuals with freezing of gait (58.7% vs 43.9%) compared to the subgroup of respondents that had used strategies to reduce arousal.

**Table 3:** Subgroup characteristics

	Have used strategies that reduce arousal	Have used strategies that increase arousal	Have used both types of strategies
Respondents (*n* (%))^a^	1122 (83.5)	92 (6.9)	98 (7.3)
Men (*n* (%))	588 (52.6)	60 (65.2)	43 (43.9)
Age (years)	67.4 ± 9.0	68.7 ± 8.6	67.8 ± 9.1
Time since diagnosis (years)	7.0 ± 5.4	7.1 ± 5.1	6.3 ± 4.9
Respondents with FOG (*n* (%))	553 (43.9)	54 (58.7)	44 (44.9)
NFOG-Q score^b^ (median [range])	18 [1–27]	17 [5–25]	17 [1–24]
Experienced ≥ 1 fall in preceding 12 months (*n* (%))	656 (58.5)	51 (55.4)	53 (60.2)

Values are presented as mean ± SD, unless otherwise specified

FOG, Freezing of gait; NFOG-Q,  New Freezing of Gait Questionnaire (score range 0–28) [29]

^a^Of respondents who had ever tried ‘Altering the Mental State’ strategies. NB: 31 respondents (2.3%) did not specify what kind of mental state strategy they had ever used

^b^Among respondents with freezing of gait, defined by a non-zero NFOG-Q score

## Discussion

Here, we present an overview of ‘Altering the Mental State’ strategies that persons with PD applied in an effort to overcome their gait impairments in daily life. Approximately one in three patients with gait impairments reported altering their mental state as a compensatory strategy. While most persons with PD reported to have used strategies that reduced arousal (e.g., by diminishing stress, or promoting general relaxation), a smaller group actually reported to have purposely increased their arousal to improve gait. We will next elaborate on our theoretical framework regarding the potential underlying mechanisms and address the clinical implications of self-modulating arousal to optimize gait performance in PD.

From a neurobiological perspective, the concept of fine-tuning one’s arousal level to optimizing one’s ability to compensate for gait impairments in PD is tightly connected to the functions of the locus coeruleus. The locus coeruleus is a small nucleus located in the posterior area of the rostral pons, and represents the primary source of noradrenaline for the central nervous system [31]. Its widespread noradrenergic projections modulate cortical, subcortical, cerebellar, brainstem and spinal cord circuits, which makes it well suited to rapidly and globally modulate brain function in response to changes in the environment (e.g., stressful stimuli) [32]. Moreover, locus coeruleus noradrenaline contributes to the reconfiguration of functional communication between distributed brain regions [33]. It is part of the ascending arousal pathway, and plays a major role in attentional and arousal response to threat [34]. Indeed, locus coeruleus activity displays an inverted u-shaped relationship with task performance, in accordance with Yerkes-Dodson’s law [35, 36]. Therefore, changes in the firing rate of the locus coeruleus may facilitate a shift in arousal that promotes optimal performance on the task at hand (Fig. 1). The neuromodulatory impact of the locus coeruleus on the central nervous system is classically compared to tuning the volume of a radio, even though its precise adaptive role is more complex and dynamic, and more appropriately analogous to the bowing of a violin [37]. Much like the effect that a bow has on the strings of a violin, the locus coeruleus changes the way the ‘notes’ are expressed (e.g., their volume or tone quality) without affecting the specific string of notes in itself, in order to shape complex neuronal melodies [37]. Increased activity of the locus coeruleus (i.e., turning up the volume) increases the strength of functional interactions between brain regions that are otherwise largely segregated [38, 39], as noradrenaline elicits changes in the internal milieu of target cells to alter their ‘neural gain’ (i.e., their excitability and receptivity to incoming signals) [40]. By mediating this increased network-level integration across the brain, the locus coeruleus can facilitate dynamic ‘cross-talk’ between different regions across the cortex and subcortex that are critical for higher-order functions [41, 42]. This functional integration is of particular importance in gait control in PD.

**Fig. 2:**
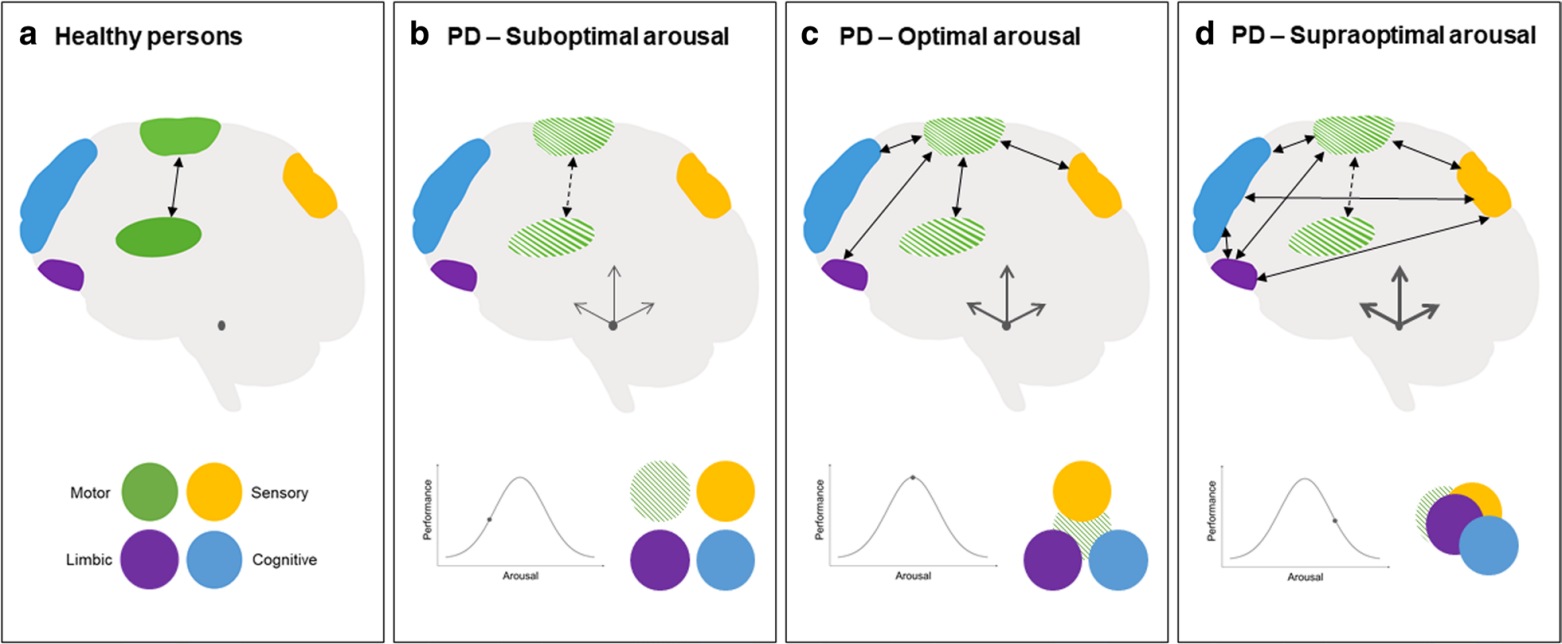
How modulating arousal may contribute to optimal gait performance in Parkinson’s disease (PD). **a** Healthy persons. In healthy persons, the primary automatic mode of motor control is intact. Different brain networks are largely segregated, as there is (usually) no need for compensatory input to achieve optimal gait control. **b**  PD—Suboptimal arousal. Impaired function of the corticostriatal motor network cannot be optimally compensated for by complementary input from other brain networks, as these networks remain largely segregated in this suboptimal state of arousal. **c**  PD—Optimal arousal. Impaired function of the corticostriatal motor network can be optimally compensated for by complementary input from other brain networks, as these networks are optimally integrated in this optimal state of arousal. **d**  PD—Supraoptimal arousal. Impaired function of the corticostriatal motor network cannot be optimally compensated for by complementary—but now competing—input from other brain networks, as these networks are engaged in dysfunctionally increased ‘cross-talk’ in this supraoptimal state of arousal. Green circle: intact corticostriatal motor network; Dashed green circle: impaired corticostriatal motor network; Yellow circle: sensory network; Purple circle: limbic network; Blue circle; cognitive network. Black double-sided triangle arrows represent a simplified schematic illustration of the functional integration between the different brain regions (which is presumably much more complex than depicted); Dashed arrows indicate the impaired function of the primary motor circuitry in PD; Thickness of the dark-grey equilateral barb arrows represents the neuromodulatory activity of the locus coeruleus (depicted here in the rostral pons as a small dark-grey ellipse). Figure inspired by Gilat et al. [48]

The pathophysiology underlying gait impairments in PD is complex and presumably involves dysfunction of multiple cortical and subcortical components. Gait partly depends on a basic ‘locomotor network’, involving spinal central pattern generators, brainstem mesencephalic and cerebellar locomotor regions, along with the corticostriatal input projecting to the primary motor cortex [43, 44]. In addition, distributed cortical areas, particularly the frontoparietal and supplementary motor areas, are normally involved in the adjustment and adaptation of walking [45]. During walking in an automated manner (i.e., without consciously paying attention to it), persons with PD typically have difficulties recruiting these cortical motor areas [46]. Adequate gait control therefore not only relies on the integrity and function of corticostriatal motor loops, but on compensatory input from cognitive, sensory and limbic systems as well (Fig. 2) [47–50]. Reinforcing the integration of these different neural networks may consequently facilitate optimal compensation for the PD-related loss of function in the motor circuitries. When locus coeruleus activity is too low (i.e., suboptimal arousal), the different compensatory networks may be too segregated to allow for adequate compensation of motor impairments. Overactivation of the locus coeruleus (i.e., supraoptimal arousal), on the other hand, may lead to a situation that allows for an element of increased, dysfunctional ‘cross-talk’ between competing inputs of these complementary networks [51]. In PD specifically, this has been associated with detrimental effects on motor function, particularly the occurrence of anxiety-induced freezing of gait [52–54]. Unfortunately, the ability to adaptively employ the locus coeruleus to optimally modulate the interaction of compensatory networks may be affected by the profound degeneration of the nucleus in PD, and this is compounded by dysfunction of cortical regions in charge of regulating arousal. Whilst well-established, this loss of noradrenergic neurons (ranging 20%–90% in PD patients [55, 56]) has been relatively neglected [57], even though it both precedes the onset and exceeds the extent of the hallmark loss of dopaminergic neurons in the substantia nigra pars compacta [58–60]. The application of ‘Altering the Mental State’ strategies to modulate arousal may therefore be necessary to facilitate shifts from sub- or supraoptimal states of arousal to optimize gait performance in PD.

Importantly, the underpinnings of these strategies presumably do not only involve noradrenaline, but other neurotransmitters such as dopamine and serotonin as well [61]. The classic parallel model suggesting a direct correlation between changes in a single neurochemical system and a distinctive deficit is likely to be too simplistic. Rather, a convergent biochemical model in which the complex interactions between the different monoaminergic systems are taken into account would be more appropriate [62]. For example, the concept of reaching a “flow state”, which respondents reported to be helpful in ameliorating gait, illustrates that striking the right balance between motivation (dopamine) and arousal (noradrenaline) can lead to optimized behavior [63, 64]. Arousal levels are also influenced by a multitude of both internal and external factors (e.g., one’s present emotions or physical environment). Even the presence of gait impairments in itself is likely to influence arousal levels in people with PD: it introduces  a vicious cycle of supraoptimal arousal due to anxiety related to falling, in turn leading to aggravated gait impairment, which further increases the fear of falling. However, while a person’s ‘baseline’ position on the curve is likely to be partly trait-dependent (e.g., a chronic state of supraoptimal arousal in those with generalized anxiety or a debilitating fear of falling), it would presumably be predominantly dependent on the task, environment and context at hand (e.g., a sudden increase in arousal when a doorbell rings). In light of this, it is unsurprising that a proportion of respondents reported to have used both types of ‘Altering the Mental State’ strategies in daily life. One can imagine that depending on the situation at hand, the state of arousal—and with that the appropriate type of strategy—may vary. Furthermore, depending on the complexity of the (gait) task at hand, the optimal level of arousal (i.e., the shape of the curve) could vary significantly across tasks [25, 65, 66]. These factors combined may make it particularly difficult to employ pharmacological interventions targeting the noradrenergic system as an add-on to dopaminergic medication to improve gait performance in persons with PD [67–70]. Any medication targeting the noradrenergic system would potentially impact the trait-dependent ‘baseline’ arousal level, rather than enabling the necessary dynamic adaptation in arousal that might be required over the course of the day. An advantage of the application of non-pharmacological compensation strategies is that it allows for such dynamic approaches that are tailored to the specific personal needs of individual patients under everchanging circumstances. Future studies could investigate whether a more personalized approach to the use of pharmacological agents targeting arousal could be beneficial in selected groups of patients (e.g., those with particularly high or low trait-dependent anxiety levels as measured by existing scales like the State-Trait Anxiety Inventory). For example, if one occupies a highly aroused state the majority of their day, could ‘resetting’ their baseline arousal level help shift them to occupy an optimal part of the curve for a greater proportion of their daily activities? Quantifying baseline levels of locus coeruleus activity using techniques such as neuromelanin-sensitive MRI [71, 72] may hold promise to stratify patients into clinical trials according to their level of noradrenergic (dys)function [73].

The survey data that we used to support our hypothesis rely on the self-reported presence of a PD diagnosis, presence of gait impairment, and the efficacy of the applied mental strategies. The potential presence of selection bias, given the selected and rather proactive subpopulation of PD patients that is typically involved in online cohorts, must also be taken into account when interpreting the survey data. However, these data were merely used to probe the presence of examples of strategies at two ends of the arousal spectrum, rather than to provide an accurate percentage of the prevalence of specific strategies. Nevertheless, future clinical trials are necessary to objectively quantify the efficacy of ‘Altering the Mental State’ strategies in improving gait performance in PD. In conjunction, measuring the effect of applying these strategies on physiological markers of arousal (e.g., measured by skin conductance, heart rate, pupil diameter) would help establish the plausibility of our proposed neurobiological framework. Lastly, looking into specific patient characteristics that may be associated with the efficacy of either arousal-reducing (e.g., high levels of trait anxiety) or arousal-increasing strategies (e.g., high levels of apathy or anhedonia) will be essential to eventually work towards a more personalized approach to the use of these non-pharmacological strategies in clinical practice [74]. Indeed, a subgroup characterization of the present cohort revealed that women less frequently reported to have used strategies to increase arousal, which may be a reflection of the higher prevalence of anxiety among women with PD [75].

## Conclusion

Our theoretical framework proposing a central role for the locus coeruleus in facilitating optimal compensation to address gait impairments provides future opportunities to investigate the control of gait, as well as guiding both targeted pharmacological (e.g., tailored use of noradrenergic agents as an add-on to dopaminergic medication in selected PD patients) and non-pharmacological therapies (e.g., a training program on ‘Altering the Mental State’ strategies for persons with PD). It is also possible that the impaired modulation of arousal arising from the dysfunction of the locus coeruleus may have consequences that extend beyond gait and could play a critical role in PD tremor, ‘wearing off’ periods, or other key symptoms that have been demonstrated to be significantly affected by one’s level of arousal (particularly in the context of stress or anxiety) [6]. Future work is necessary to validate this conceptual framework, quantify the efficacy of ‘Altering the Mental State’ strategies, and further crystallize the potential involvement of the noradrenergic system in optimizing motor performance in PD.

## Data Availability

All individual data collected through the Fox Insight online clinical study are available on the Fox Insight database (https://foxinsight-info.michaeljfox.org/insight/explore/insight.jsp). Qualified researchers, interested in PD or related research, can access Fox Insight data upon account registration, completion of a Data Use Agreement (DUA), acknowledgement of the study publication policy, and review by the Data and Publications Committee. Other data related to the present work are available upon request to the corresponding author.

## Abbreviation

PD: Parkinson’s disease
